# Proteolytic Remodeling of the Synaptic Cell Adhesion Molecules (CAMs) by Metzincins in Synaptic Plasticity

**DOI:** 10.1007/s11064-012-0919-6

**Published:** 2012-11-04

**Authors:** Malgorzata Bajor, Leszek Kaczmarek

**Affiliations:** Laboratory of Neurobiology, Nencki Institute, 3 Pasteur Street, 02-093 Warsaw, Poland

**Keywords:** Proteolysis, Synapse, Dendritic spines, MMP, SynCAM, Cadherin

## Abstract

Cell adhesion molecules participate in the formation, maturation, function and plasticity of synaptic connections. The growing body of evidence indicates that in the regulation of the synaptic plasticity, in which these molecules play pivotal role, also the proteolytic processes are involved. This review focuses on extracellular proteolysis of the cell adhesion molecules by specific subgroup of the matrix metalloproteinases, a disintegrin and metalloproteases and a disintegrin and metalloproteinase with thrombospondin motifs, jointly referred to as metzincins, in driving coordinated synaptic structural and functional modifications underlying synaptic plasticity in the adult brain.

## Introduction

After the initial establishment of neuronal connections during development, synapses remain highly dynamic and undergo activity-dependent changes in their efficacy and morphology. Communication between neurons at the synapses is mediated primarily by neurotransmitter release and by the gating of the postsynaptic receptor ion channels, but burgeoning evidence indicates that signaling is also mediated by adhesion molecules that interact in a homo- or heterophilic fashion across the synaptic cleft. Thus, cell adhesion molecules (CAMs) at the synapse mediate synaptic plasticity, the ability to change synaptic function, thought to underlie learning and memory, as well as implicated in a number of neuropsychiatric conditions. It is, however, still poorly understood how synaptic CAMs contribute to synapse formation and/or structure, and whether and/or how smaller groups of CAMs serve as minimal, functionally cooperative adhesive units upon which the structure is based [1].

An increasing number of studies support the idea that structural changes at the synapses are closely associated with synaptic plasticity. A majority of these dynamic changes in the synaptic microenvironment are regulated by various families of proteases, including mainly metzincins [2] and serine proteases. Their function is to cleave the proteins available in the extracellular matrix (ECM) and even to release signaling molecules from ECM and CAMs, which may play an essential role for changes in synaptic configuration. Notably, ECM remodeling affects both structural and functional plasticity, such as long-term potentiation (LTP), long-term depression (LTD), homeostatic plasticity, and metaplasticity [3]. Importantly, the synaptic remodeling involves a complex sequential proteolytic activation and interaction of different molecules resulting in the control of various processes acting at the synapse, such as receptors trafficking, cytoskeleton remodeling, formation, growth and morphological changes of new and existing dendritic spines. Notably, it has repeatedly been shown that the same target molecule can be recognized and processed by various proteases and the released soluble ectodomains of CAMs may interact with different ligands, leading to the generation of distinct signals.

## Proteolysis of Cell Adhesion Molecules

At the synapse, cell adhesion molecules operate in synergistic association in the control of adhesive function and signal transduction by forming dynamic network rather than acting as independent molecules. Similarly, the processes associated with remodeling of neuronal connections are achieved by the concerted actions of several different proteases that are secreted by neurons and glial cells [4, 5]. One of the most prominent features between CAMs and proteases actions is that they are reciprocal. Metzincins are apparently the major effectors in remodeling the structures of neuronal circuits which contribute to the fine tuning of diverse biological processes through limited proteolysis of specific targets. Recent data strongly imply their important role in the modulation of morphology of dendritic spines which lead to changes in signaling pathway and molecules trafficking in the brain. Table 1 contains summary information on cleavage of CAMs by metzincins and the role of their proteolytic processing in synaptic plasticity.

**Table 1:** Metzincins implicated in synaptic plasticity and their substrates in the brain

Family	Synaptic substrates	Metzincin cleavage	Possible synaptic effects of processing	Refs.
Cadherins	N-cadherin	MT5-MMP, ADAM-10	Proliferation of neuronal stem cells, neuronal differentiation, axonal remodeling and synaptogenesis, modulation of dendritic spine morphology	[7–10]
Syndecans	Syndecan-1	MMP-2, -7, -9, MT1-MMP, MT3-MMP, ADAM-17	Dendritic spine development, cell adhesion, neurite guidance, cell migration during development of CNS	[25, 26, 28–33]
	Syndecan-2	MMP-2, -9		
	Syndecan-4	MMP-2, -9, ADAM-17, ADAMTS-1, -4		
Immunoglobulin superfamily	NCAM	MMP-2, ADAMs	Neurite branching and outgrowth, influence on LTP, structural remodeling of neuronal connections	[37, 38, 46, 47]
	L1-CAM	ADAM-10, -17	Neurite outgrowth, neuronal differentiation and migration	[41]
	Nectin-1	ADAM-10	Regulation of the spine density and morphology, cell–cell interaction, influence on LTP and LTD	[49–51]
	SynCAM 1	ADAM-17	Modulation of synaptic interactions	[51]
	SynCAM 2	MMP-9	Remodeling of dendritic spine structure	[56]
	ICAM-5	MMP-2, -9	Maturation of dendritic spines, cytoskeleton reorganization, morphological remodeling of dendritic spines,	[57, 62, 63]
Dystroglycans	β-dystroglycan	MMP-9	Cytoskeleton reorganization, modulation of dendritic spine morphology	[65, 66]
Neurexins	β-neurexin	MMPs	Regulation of the synapse formation and stabilization of the connectivity in the CNS	[69, 70]
Thrombospon-dins	TSP1	MMP-2, ADAMTS1	ND	[74–79]
	TSP2	MMP-2, -9, -14, ADAMTS1		
	TSP5	MMPs		

*ND* not defined

## Cadherins

Among the principal representatives of the CAMs are cadherins. Members of the cadherin superfamily share general expression profiles and have distinct functions during the brain development and in the mature brain function. Their main role is to tie up the pre- and postsynaptic part of synapse by homophilic interactions. Inside the cells, cadherins are bound to the actin cytoskeleton via β-catenins and affect synaptogenesis, maintenance of cell–cell contact and dendritic spine morphology [6]. It was shown that surface population of cadherin molecules is regulated by proteolytic cleavage in the extracellular matrix or intracellular milieu. Neuronal cadherin (N-cadherin) undergoes the ectodomain cleavage by at least two membrane-bound matrix metalloproteinases (MMPs), ADAM10 and membrane type 5-matrix metalloproteinase (MT5-MMP), followed by the intramembrane proteolysis mediated by PS1/γ-secretase complex [7–10]. ADAM10 plays a crucial role in the complex sequence of events that regulates dendritic spine maturation and/or stabilization and in the modulation of the structural organization of the glutamatergic synapse [7]. The ADAM10 driven processing in response to calcium influx via NMDA type glutamate receptor is required for the subsequent PS1/γ-secretase cleavage of N-cadherin, which leads to releasing of a cytoplasmic fragment. This process is called regulated intramembrane proteolysis (RIP), in which removal of the ectodomain by shedding is necessary for the second cleavage catalyzed by γ-secretase [11]. The proteolytic processing of N-cadherin affects the interaction between N-cadherin and β-catenin, leading to release of β-catenin from the cell surface and directing it to the nucleus, to regulate genes involved in the proliferation of neuronal stem cells, neuronal differentiation, axonal remodeling and synaptogenesis [9]. It might be suggested that such proteolytic modulation of β-catenin interaction with N-cadherin may be a substantial molecular mechanism for the synaptic plasticity.

Recently, Gardoni et al. [12] have shown that PACAP38 has an impact on the modulation of dendritic spine morphology through the ADAM10-N-cadherin-AMPA receptor signaling pathway. Pituitary adenylate cyclase-activating polypeptide 38 (PACAP38) is neuropeptide that is implicated in the induction of the synaptic plasticity at the excitatory glutamatergic synapse. It has been shown that PACAP38 induces a form of long-term depression in the hippocampal neurons and transgenic animals, thus indicating its role in learning and memory [13–16]. Moreover, PACAP38 leads to a strong increase of ADAM10 activity through three signaling cascades (PKC, MAP kinase and PI 3-kinase) [17]. Impairing ADAM10 localization and activity at the synapse decreases its processing of N-cadherin. Thus, it induces a modification of the synaptic AMPA receptors, and a significant increase in the size of dendritic spines, both in vitro and in vivo [7].

More recently, Warren et al. [18] have shown the differences in the expression and distribution of ADAM10, MT5-MMP and N-cadherin after traumatic brain injury (TBI), leading to maladaptive synaptic plasticity. MT5-MMP and ADAM10 are critical to the success of injury-induced synaptic plasticity. They displayed time-dependent increases during synaptogenesis, and elevated enzyme activity was concatenated with a reduction of the synaptic adhesion protein, N-cadherin.

ADAM10 inhibition contributes to elevated N-cadherin expression and improves synaptic stability under maladaptive conditions. Sustained N-cadherin expression is reported to underlie both structural and physiological plasticity at the synapse [6, 19, 20]. Furthermore, it was also shown that manipulation of cadherins by either using specific blocking peptide or antibodies, or genetic mutations lead to disruption of N-cadherin molecule that has a profound and long-lasting effect on synaptic plasticity and memory formation [19, 20]. Moreover, a dysfunction of the cadherin-based adhesive system may alter functional connectivity and information processing in the human brain in neuropsychiatric disorders [21].

Notably, the ectodomain shedding of other members of cadherin family in multiple pathophysiological conditions, including inflammation and cancer, has been observed. For instance, it was demonstrated that vascular endothelial cadherin (VE-cadherin), an endothelial-specific member of cadherin family, that forms a complex with beta-catenin and stabilizes cell–cell adhesion is also cleaved by MMP-7 on the cell membrane of the human umbilical vein endothelial cell. This phenomenon might play an important role in angiogenesis [22]. Moreover, ADAM10-mediated endothelial cadherin (E-cadherin) proteolysis was proposed as a regulatory mechanism in inflammatory epidermal diseases, which are characterized by loss of E-cadherin expression and loss in epithelial integrity [23]. In breast cancer, it was observed that upregulation of the ADAM15 leads to enhance cleavage of E-cadherin [24]. In the brain, during early stage of neural development the presence of E-cadherin has been observed. It was shown that E-cadherin is first expressed in the embryonic ectoderm and plays a role in maintaining of ectoderm and future epidermis architecture together with other cadherins. Soon after neural induction, E-cadherin is replaced by N-cadherin. So far, however, it has apparently not been shown that E-cadherin is processed by metzincins in the brain.

## Syndecans

Syndecans are a major class of heparin sulfate proteoglycans (HSPGs) and are involved in adhesion-induced synaptic modifications. Four members of the syndecan family, syndecan-1, -2, -3, and -4, have been characterized in the mammalian tissues, including the brain. For instance, syndecan-2 plays a prominent role in the organization of postsynaptic structures. It is highly concentrated on the spines of mature hippocampal neurons, and plays a critical role in the dendritic spine development [25]. The other member, syndecan-3, is involved in cell adhesion, neurite guidance, and cell migration during development of the nervous system [26]. Moreover, it has been suggested to function as an important modulator of the synaptic plasticity that influences hippocampus-dependent memory [27]. Through interaction with several cytoplasmic proteins, syndecans may provide a molecular link between intracellular cytoskeleton/signaling complex and the extracellular environment at specific sites on the cell surface [25].

It has been shown that syndecans undergo regulated proteolytic shedding from the cell surface. Interestingly, syndecans are differentially cleaved by three classes of metzincins: MMPs, a disintegrin and metalloproteases (ADAMs) and a disintegrin and metalloproteinase with thrombospondin motifs (ADAMTS) proteases. MMP-2 and MMP-9 can cleave syndecans-1, -2 and -4 [28, 29]. MMP-7 and membrane-associated metalloproteinases MT1-MMP and MT3-MMP are known to cleave syndecan-1 [30, 31]. It has recently been demonstrated that ADAM17 is able to shed syndecan-1 and syndecan-4 [32], and the latter is also processed by ADAMTS1 and ADAMTS4 proteases [33]. Moreover, for it has been shown that the remaining portion of the syndecan-3 can be further processed by the PS1/γ-secretase complex. The multiplicity of proteases able to cleave syndecans may result from the heterogeneity of their ectodomains and may depend on the cell type and stimulatory conditions. Hence, still is not clear how extracellular stimuli influence sheddases to mediate syndecan cleavage. Although, the syndecans cleavage was not demonstrated in the brain, it cannot be excluded that some of their neuronal functions may be regulated by proteolysis.

## NCAM and L1-CAM

NCAM and L1 are members of immunoglobulin-like superfamily that were repeatedly implicated in synaptic functions, as well as neuronal migration, neuronal survival, neurite outgrowth, myelination, axon guidance, fasciculation, and regeneration [34–36].

Both NCAM and L1 were shown to be processed by metzincins. Inhibition of NCAM-mediated adhesion with either function-blocking antibodies or synthetic peptides did not affect normal basal synaptic transmission, but reduced E-LTP in area CA1, along with impaired hippocampal-dependent learning [37]. Hinkle et al. [38] revealed that regulated ADAM metalloprotease-induced ectodomain shedding of NCAM down-regulates neurite branching and neurite outgrowth in primary cortical neurons.

In vitro and in vivo studies showed that cleavage of L1 protein is elicited by two sheddases ADAM10 and ADAM17. Both proteases critically affect the physiological functions of L1 adhesion protein. Proteases-mediated disruption of L1-dependent contacts might be an important mechanism for the regulation of the adhesion of migrating neurons. Moreover, ADAM10-dependent releasing of soluble L1 ectodomain from cultured neurons promotes neurite outgrowth and influences neuronal differentiation. In addition, it was shown that NMDA-stimulated Ca^2+^ influx might be the cause for enhanced ADAM10 activity, leading to increased L1 shedding. This Ca^2+^ influx is known to affect activity-dependent synaptic plasticity [39]. Besides, L1 is also cleaved by PS1/γ-secretase complex. This regulated intramembrane proteolysis process [40], also demonstrated for the N-cadherin or nectin-1, affects signal transduction at the synapse. Furthermore, Matsumoto-Miyai et al. [41] found that the plasticity-related L1 is a specific substrate of neuropsin and that this neuropsin-L1 processing system is regulated by neural activity and is involved in the hippocampal plasticity.

NCAMs are involved in the initial phase of long-term potentiation in the hippocampus and learning [42, 43]. It has been reported that NCAM may be cleaved extracellularly in vivo in response to the activation of NMDA receptors during the induction of LTP and in response to seizures [44, 45]. Additionally, Hubschmann et al. [46] have shown that NCAM can be released from the primary hippocampal neurons in vitro and this cleavage involves extracellular ATP and can be inhibited by the metalloproteinase inhibitor. The ATP released either during learning or induction of LTP may target NCAM for proteolysis, and this proteolysis is necessary for the structural remodeling of neuronal connections taking place during consolidation of LTP. All these findings suggest that metalloproteinase activity regulates NCAM-mediated neurite outgrowth, possibly by cleaving NCAM from the extending neurite, thereby reducing adhesion to an immobile environment and thus facilitating further neurite extension [46]. The members of NCAM family are also implicated in aberrant plasticity. It was shown that neural cell adhesion molecules and metzincins, together play an important role in the pathogenesis of experimental autoimmune encephalomyelitis (EAE). The observed elevated level of MMP-2 and impaired expression of NCAM in the hippocampus appear to be critical for both the brain plasticity and underlie a complex autoimmune process in the brain in acute EAE [47].

The expression of the paralog of NCAM, NCAM2 has been proposed to influence certain types of neurological diseases and cancers. Both proteins are abundant in the central nervous system (CNS), suggesting that they also may share functional similarities. To date, there is no information about proteolytic cleavage of NCAM2. Nevertheless, it cannot be excluded that in the olfactory system, where NCAM2 is important for the formation or maintenance of the dendritic and axonal compartmentalization, its function might be regulated by proteolysis, similarly to NCAM1 [48].

## Nectins

Nectins are Ca^2+^-independent immunoglobulin-like adhesion molecules, involved in cell–cell adherent junctions. Nectin-1 ectodomain shedding and intramembrane cleavage occurs in postsynaptic as well as presynaptic membranes, where it is localized [49, 50]. Kim et al. [51] have shown that nectin-1 cleavage plays a role in the regulation of the spine density. It is well documented that activation of NMDA receptors or chemical long-term potentiation (cLTP) result in ADAM10 activation which is one of the major proteases responsible for ectodomain shedding of nectin-1 in neurons [49, 51]. The extracellular proteolysis of nectin-1 generates at least two soluble ectodomains [50]. These protein fragments may act as signaling molecules in the synaptic cleft by interacting with other ECM components and thus regulating cell–cell interaction or may bind to their receptors, initiating a cascade of signaling inside the cell. Thus, taken together, these events may influence on changes in density and spine morphology observed during induction of LTP and LTD. On the other hand, the intramembrane domain of nectin-1 released by activity of presenilin-dependent gamma-secretase complex may also serve many roles. The *C*-terminal fragment of nectin-1 may be translocated into the nucleus and acts as either transcriptional stimulator or repressor. Sequence analysis revealed that this intracellular fragment contains a putative nuclear localization signal (RRRH) right after the transmembrane domain [52]. Moreover, cleavage of nectin-1 releases cytoplasmic proteins such as afadin from peripheral membranes and causes its translocation into the nucleus and thus, may regulate the subcellular localization of afadin between the plasma membrane and the nucleus [53].

## SynCAMs

Synaptic cell adhesion molecules (SynCAMs) belong to the immunoglobulin-like protein family and act as an adhesion molecules in the synaptic cleft forming a homo- and heterophilic transsynaptic adhesion complexes that contributes to synapse organization and function [54]. Tanabe et al. [55] have shown that SynCAM1 can be processed by ADAM17-like proteases at the synapse. The biological importance of this phenomenon may be associated with the modulation of synaptic interaction and plasticity. Recently, using proteomic approach, the cleavage of SynCAM2 via MMP-9 has been demonstrated [56]. SynCAM2 cleavage evoked by MMP-9 might influence the remodeling of the dendritic spine structure in response to synaptic transmission. It indicates that different SynCAMs can be cleaved by various types of proteases, however, the exact mechanism of this process still remains unknown.

## ICAM-5

Tian et al. [57], showed that intercellular adhesion molecule-5 (ICAM-5), protein specifically expressed in postnatal excitatory neuronal cell bodies, dendritic shafts, and dendritic filopodia of the telencephalon [58, 59], is a substrate for either MMP-2 or MMP-9. Upon neuronal stimulation, ICAM-5 cleavage driven by MMPs, caused the dendritic spines maturation and elongation of filopodia. Moreover, blocking of MMP-2 and MMP-9 by specific inhibitor as well as ICAM-5 deficiency led to the retraction of the spine heads and a decreased number of spines in response to NMDA stimulation [57]. The mechanism of spine remodeling through MMPs dependent ICAM-5 cleavage has been proposed [60, 61]. In neurons, stimulation of either NMDA or AMPA receptors for glutamate leads to enhancement of MMP-2 and MMP-9 activity, resulting in processing of ICAM-5 from immature nascent spines. The shedding of ICAM-5 may facilitate local membrane and cytoskeleton reorganization, and thereby morphological remodeling of the dendritic spines [57]. In addition, recent results have shown that ICAM-5 can be also cleaved by exogenous MMP-3 and MMP-7, in response to neuronal activation by either NMDA treatment or induction of LTP [62].

Furthermore, it was shown that soluble ICAM-5 is generated with cLTP. The *N*-terminal domain of ICAM-5 is able to stimulate integrin dependent actin polymerization within dendrites, and thus spine expansion. In addition, the inhibition of MMPs activity and blocking of β1 integrins diminished ICAM-5 dependent effects. Overall, these findings indicate that MMPs and soluble ICAM-5 have the potential to influence neuronal excitability [63].

## β-Dystroglycan

Dystroglycan is a part of dystrophin-glycoprotein complex that links dystrophin and the intracellular cytoskeleton with extracellular matrix and anchors the whole complex at the membrane [64]. DG is composed of α- and β-subunits. α-dystroglycan (α-DG) is a highly glycosylated extracellular component, whereas β-DG spans the plasma membrane forming a bridge between α-DG and the cytoskeleton [65]. Michaluk et al. [66] have shown that MMP-9 driven β-DG proteolysis occurs in response to synaptic activity in neuronal cultures and in the hippocampus in response to seizures. Simultaneously, in neurons, after treatment with specific inhibitor (TIMP-1) blocking MMP-9 activity and in MMP-9 knockout mice, the appearance of truncated form of the protein was not observed. So far, the exact consequences of β-dystroglycan cleavage are not known, however, a growing body of evidence suggests a functional role for the entire dystrophin-glycoprotein complex at central synapses and in their plasticity. For instance, it was shown that mice selectively deficient in the brain dystroglycan suffer from late phase of long term potentiation deficits in the hippocampus [65]. Moreover, mutations in the genes encoding dystroglycan-binding proteins such as laminin, its extracellular ligand, as well as dystrophin, are associated with mental retardation. Notably, specific changes in the hippocampal expression patterns of transcripts encoding dystrophin and neurexins, presynaptic interacting partners of DG, following kainate and PTZ treatment in vivo have been previously demonstrated [67, 68].

## β-Neurexin

It was also established that cell surface adhesion receptors undergo proteolytic cleavage in the synaptic cleft. Among them, it was shown that biological role of β-neurexin is closely associated with its proteolytic processing. The presynaptic receptors neurexins (NRXs), and their ligands, postsynaptic neuroligins (NLs) are two families of synapse-specific adhesion molecules critically involved in regulation of the synapse formation and stabilization of the connectivity in the central nervous system. It was demonstrated that mutations in neurexins may lead to synaptic defects associated with brain disorders (e.g. mental retardation, autism). Studies by several independent groups have shown that neurexins can be proteolytically processed at the synapse [69, 70]. Thus, both in vitro and in vivo neurexins are sequentially cleaved by metalloprotease- and PS1/γ-secretase dependent activities. In neurons, accumulated *N*- and *C*-terminal fragments of β-neurexin play a dual role, as signaling molecule in the extracellular milieu as well as in the intracellular space.

## Thrombospondins

Thrombospondins (TSPs) are involved in cell–cell interactions and synaptogenesis and have the ability to bind to the matrix proteins, proteinases, growth factors, and cell surface receptors. Recently, it was shown that thrombospondin 1 and thrombospondin 2 promote synaptogenesis, both in vitro and in vivo, and their deficiency results in reduced synaptic density during development [71]. Moreover, both of them are involved in modulation of the synaptic plasticity and axonal sprouting after ischemic injury [72]. However, one of the best described roles of TSP1 is action as an anti-angiogenic agent. TSPs functions have been well established in cancer biology where angiogenesis is essential for tumor growth [73].

To date, many reports indicate that TSPs are processed by various proteases in extracellular matrix. It was shown that ADAMTSs are involved in the processing of extracellular glycoproteins, including TSP1 and TSP2 [74]. For instance, cleavage of TSP1 and TSP2 by ADAMTS1 could modulate their functions by facilitate remodeling of matrix-associated TSP [75]. Furthermore, most recently, the global proteomic studies discovered that TSPs are also targets for other metzincins. Hence, thrombospondin-1 and thrombospondin-2 are MMP-2 [76] and MMP-14 [77] substrates, respectively. Moreover, for thrombospondin-2 the cleavage by MMP-2 and MMP-9 was confirmed [78]. TSP5 was also shown to be proteolytically processed by different MMPs in vitro [79]. Nevertheless, despite the established role of TSPs in the brain, especially during synaptogenesis, so far, there is no direct data about the regulation of these proteins by proteolysis.

## Future Directions

Interest in the identification of proteodegradome in the extracellular matrix, including discovering of the new targets for proteases and understanding their function, has intensified in recent years in a hope to increase our knowledge of the synaptic machinery. In this review, we emphasize that cell adhesion molecules play not only scaffolding role in the synaptic cleft, but foremost are capable to modulate the functional and structural aspects of the synaptic plasticity in both normal and pathological conditions. It has to be noted that presented list of CAMs which undergo proteolytic turnover at the synapse is still open. Completion of genome sequences for many organisms allows to protein classification into the respective clusters. Based on individual protein domain structures for extracellular matrix proteins the ECM and adhesion proteins, the concepts of “matrisome” and “adhesome” have been implemented [75, 76]. This opens a window for classification of new ECM and ECM-associated proteins as important in regulation of the synaptic plasticity and understanding how these components can influence neuronal network dynamics.

## Abbreviations

PS1/γ-secretase: Presenilin 1/gamma-secretase
NMDA: N-Methyl-d-aspartic acid
cLTP: Chemical long term potentiation
PKC: Protein kinase C
MAP kinase: Mitogen-activated protein kinase
PI 3-kinase: Phosphoinositide 3-kinase
PTZ: Pentylenetetrazol
